# The Effectiveness of Acupoint Catgut Embedding Therapy for Abdominal Obesity: A Systematic Review and Meta-Analysis

**DOI:** 10.1155/2019/9714313

**Published:** 2019-06-23

**Authors:** Jili Sheng, Xiaoqing Jin, Jianfang Zhu, Yidan Chen, Xu Liu

**Affiliations:** Department of Acupuncture, Zhejiang hospital, Hangzhou 310013, China

## Abstract

Acupoint catgut embedding is a useful therapy for weight management and widely applied in China. This review aimed to systematically evaluate the effects of acupoint catgut embedding on abdominal obesity. We searched the PubMed, Cochrane-Library, Embase, OVID, MEDLINE, ISI (web of science), Wanfang, VIP, CBM, and CNKI for randomized controlled trials that used acupoint catgut embedding to treat abdominal obesity before April 2019 with the language restriction of Chinese and English. The combination subject terms of abdominal obesity (or central obesity) and acupoint catgut embedding (or catgut implantation, catgut embedding) were used. We found 15 studies involving 1584 individuals. When acupoint catgut embedding plus electroacupuncture is compared with electroacupuncture alone, significant reductions in improvement rate (RR = 1.03, 95% CI = 0.99~1.08), body weight (MD = 5.20, 95%CI = 1.16~9.25), body mass index (MD = 1.73, 95%CI = 0.70~2.76), waist circumference (MD = 2.91, 95%CI = 1.36~4.46), and hip circumference (MD = 1.06, 95%CI = -0.18~2.30) were found. Mean values of body weight by acupoint catgut embedding were 1.35 kg compared with electroacupuncture. Less adverse events were reported in all included articles. In summary, pooled outcomes of acupoint catgut embedding presented a tendency of equal effects to other kinds of acupuncture, whereas acupoint catgut embedding plus electroacupuncture is more effective for abdominal obesity. This study is registered with PROSPERO 2017 (CRD42017082357).

## 1. Introduction

Obesity is an increasingly prevalent health problem that is related to various diseases, such as diabetes mellitus, cancer, and cardiovascular diseases [1]. Abdominal obesity, a special kind of obesity, was considered to be more associated with the disease compared with non-abdominal obesity [2]. It is usually manifested as extravagant abdominal fat around the stomach and abdomen leads to a negative influence on health. Currently, absolute WC (>102 cm (40 in) in men and >88 cm (35 in) in women) [3] and the waist-hip ratio (>0.90 for men and >0.85 for women) are conventional methods for diagnosis [4]. In the previous study, abdominal obesity was linked to altered reward and cognitive systems which regulate the appetite response [2]. Recently, many studies have been performed to prove the strong associations between abdominal obesity and metabolic syndrome which is linked to diabetes and metabolic syndrome [5, 6]. Salehinia et al. have assessed the relationship between abdominal obesity and a higher risk of incident diabetes successfully [7]. The strong correlation between abdominal obesity and cardiovascular disease also has been proved. Thus, it has been seen that people with abdominal obesity have a high risk of metabolic syndrome [5].

Options for treatment of abdominal obesity are much similar, including lifestyle intervention, surgery, and drug. But considering the high risk of pharmacotherapy and surgery, more clinicians have applied traditional Chinese medicine (TMC) for weight control [8], such as manual acupuncture (MA), electroacupuncture (EA), and acupoint catgut embedding (ACE). Acupuncture can promote weight control by regulating the nervous, endocrine and digestive system [9]. An analysis in 2011 found that acupuncture and drugs had better efficacy than lifestyle intervention, and acupuncture had fewer reported adverse effects [10]. The electroacupuncture and ACE were a combination of acupuncture and modern technologies. Now more and more researchers have focused on the overall effect and mechanism of electroacupuncture and ACE on weight loss. The principle of electroacupuncture is that a small electric current is passed between pairs of needles. Gong et al. found electroacupuncture had a good influence on weight loss through the regulation of AMPK signaling pathways [11, 12]. ACE is an integrative medicine with some absorbable catgut sutures implant into the acupoint based on the theory of acupuncture. The advantages of ACE are easy operation and durable stimulation compared with MA and EA [13].

Several comparative analyses have been performed to investigate the curative effect of MA, EA, and ACE. A meta-analysis in 2015 found that ACE presented a better outcome compared with MA and EA for obesity [14]. However, to the best of our knowledge, there was no comparative analysis focusing on the curative effect of the above three therapies for abdominal obesity.

The present meta-analysis aims at systematically evaluating the evidence on the effects of ACE therapy for weight management of abdominal obesity patients. We searched electronic databases to obtain relevant studies published before April 2019 with restriction of Chinese and English language. The forms of acupuncture included MA, EA, and ACE in this research.

## 2. Methods

### 2.1. Data Sources and Search Strategy

A comprehensive literature search was performed from the initiation to April 2019 in the database of PubMed, the Cochrane Library, Embase, OVID, MEDLINE, ISI (web of science), and four Chinese databases, including CNKI, VIP, Wanfang, and CBM. Combination subject terms of abdominal obesity (or central obesity) and acupoint catgut embedding (or catgut implantation, catgut embedding) were used to search randomized controlled trials (RCTs), and the languages of Chinese and English were restricted.

### 2.2. Study Selection, Inclusion and Exclusion Criteria

Eligible studies were identified by reading the titles and abstract of retrieved database literature, according to the following inclusion and exclusion criteria. Disagreements were resolved by a third if necessary.

These studies included in the meta-analysis met the following criteria: (1) the study design must be a clinical RCTs with ACE (or ACE plus control) as treatment group and MA (or EA or diet) as control; (2) patients diagnosed with abdominal obesity irrespective of ages and sex as study subjects; diagnostic criteria must be clear, and inclusion and exclusion criteria were explicit; (3) English and Chinese language studies.

The following were excluded: (1) obesity patients diagnosed with other diseases; (2) studies that compare the effect of the difference of catgut length, operation, or acupoint prescription; (3) duplicate publications and studies with the same results.

### 2.3. Data Collection and Quality Assessment

The extracted information included main characteristics of included RCTs (i.e., authors, publication year, and location), characteristics of participants (i.e., age, gender, BMI, and sample size), details of the intervention, and type of outcomes. The methodological quality of eligible literature was assessed using the methods recommended in the Cochrane Handbook for Systematic Reviews of Interventions [15]. The literature was ranked high, low, and unclear risk by using the Cochrane Handbook V.5.1.0 to evaluate the bias risks of each included RCT from the aspects of random sequence generation, allocation concealment, blinding of outcome assessment, incomplete outcome data addressed, and selective outcome reporting.

### 2.4. Statistics Analysis

Review Manager (version 5.3, the Nordic Cochrane Center, Copenhagen, Denmark) was applied to assess curation effect and publication bias. The relative strength of curation effect was illustrated by forest plot. Heterogeneity among RCTs was assessed using the Chi^2^ and* I*^2^ statistic. Random effect model was chosen owing to the potential clinical heterogeneity in different trials resulting from the different acupoints applied in different catgut embedding therapies. The relative risk (RR) and mean difference (MD) with 95% confidence interval (CI) were used to analyze continuous data.* P* < 0.05 was considered statistically significant.

## 3. Results

### 3.1. Study Description and Participants

**Figure 1:**
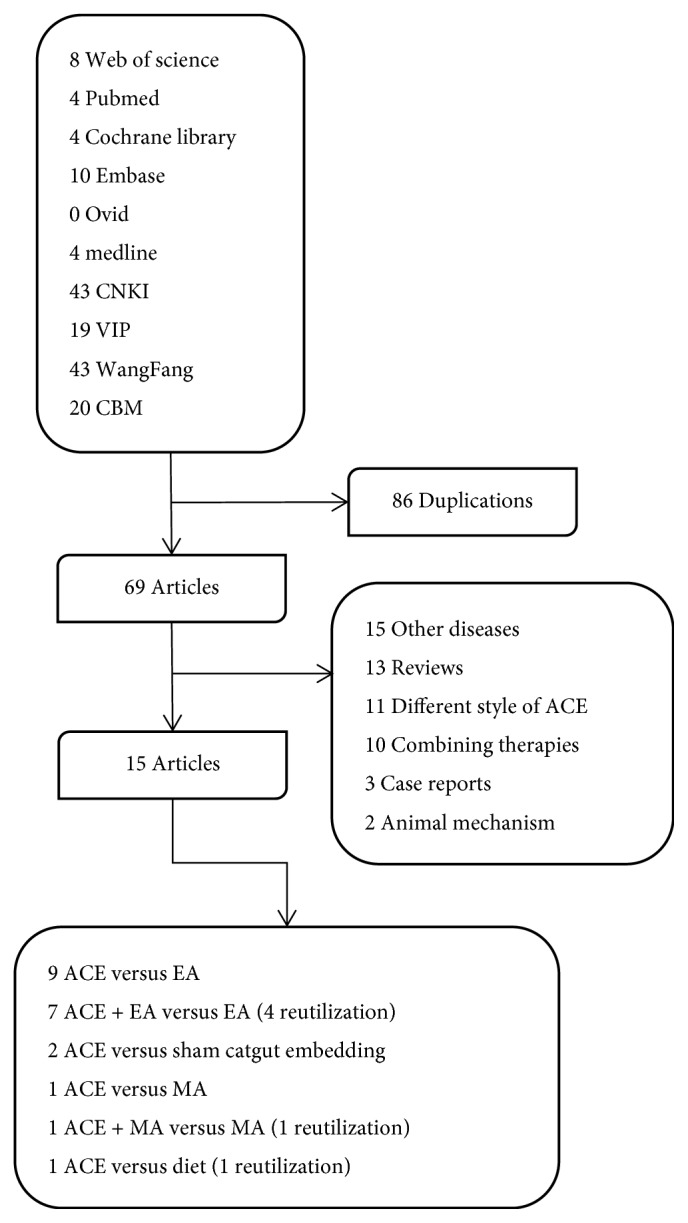


Our initial search obtained 155 potentially papers from the databases, of which 70 were reserved with 85 excluded for duplication. 15 articles were selected after screening the titles and abstracts based on the inclusive and exclusive criteria. Finally, we included 15 studies with 1584 participants [13, 16–29]: [13, 21] in English and [16–20, 22–29] in Chinese met the inclusion criteria. Among these studies, there were 9 articles [17, 22–29] that reported the weight loss effect of ACE (402 patients) with EA (401patients), 7 ACE plus EA (345 patients) versus EA (345 patients) [18–20, 23, 25, 27, 28], 2 ACE (103 patients) versus sham (99 patients) (that with the same operation as ACE but the catgut was not implanted) [16, 21], and 1 article [13] performed the comparative analysis of ACE versus MA, ACE plus MA versus MA and ACE versus diet with 30 patients for each group. The articles were filtered as shown in Figure 1.

### 3.2. Risk Bias in Included Studies

**Figure 2:**
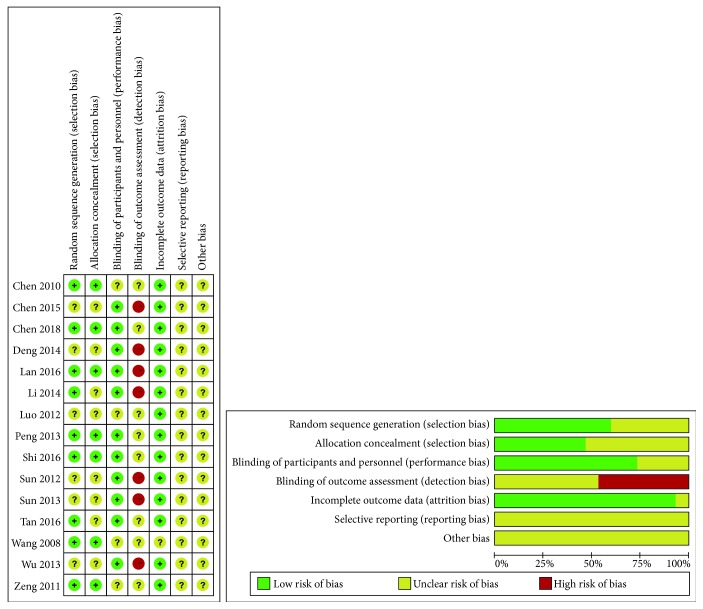


The methodological quality of all included studies was poor and probably at high risk as shown in Figure 2. Of the 15 studies, 9 [16–18, 20, 21, 23, 24, 28, 29] reported the random sequence generation and 7 [13, 22, 25–27] reported the blinding of outcome assessment in high risk. The sample size varied from 30 to 100 participants. Two articles [16, 17] reported a small proportion of dropout whose data were also excluded from the analysis. The basic characteristics of included trials were summarized in Table 1. The selective reporting of outcomes cannot be judged without published trial protocols or registration of included studies.

### 3.3. Comparison: ACE versus EA

#### 3.3.1. Frequency of Improvement

**Figure 3:**
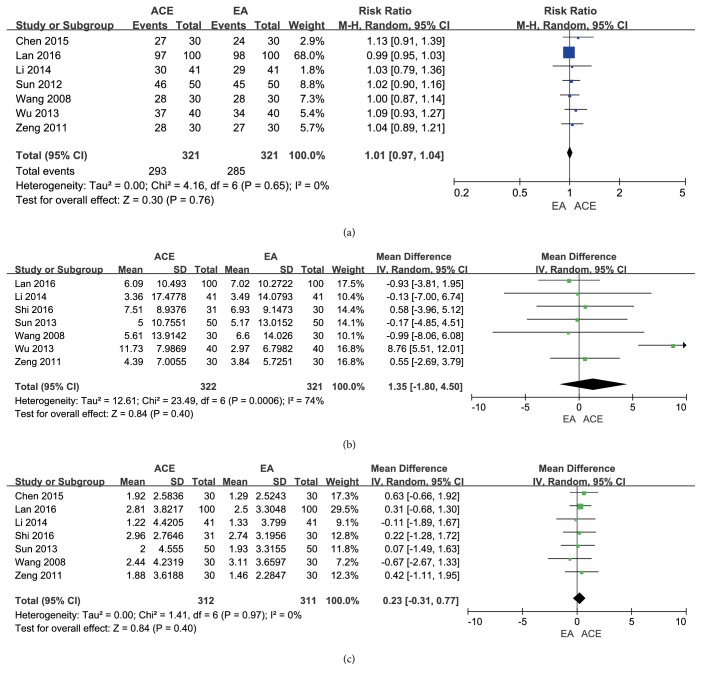


There were 9 trials with 803 patients [17, 22–29] in the comparison of ACE versus EA, of which 7 trials [22–24, 26–29] with 642 patients evaluated the frequency of improvement. The low heterogeneity was detected (*I*^2^ = 0%, Chi^2^ test* p* = 0.65), and the random effect model was applied to calculate the incorporated data. The results showed no difference in improvement rate between the two groups (RR = 1.01, 95% CI = 0.97~1.04,* p* = 0.76) (Figure 3(a)).

#### 3.3.2. Reduction of BW and BMI

Seven meta-analyses [17, 23–25, 28, 29] that compared the outcome of the BW loss for abdominal obesity patients between ACE and EA treatment showed no significant difference between the groups (MD = 1.35, 95%CI =-1.80~4.50,* p*=0.40). The random effect model was used owing to their statistic heterogeneity (*I*^2^ = 74%, Chi^2^ test* p* = 0.0006) which might be caused by the differences of the frequency of intervention, manipulations, and participants (Figure 3(b)).

For the decline of BMI, as shown in Figure 3(c), the merged results of 7 studies [17, 22–25, 28, 29] demonstrated no variance between the two groups (MD = 0.23, 95%CI = -0.31~0.77,* p* =0.40). No significant heterogeneity (*I*^2^ = 0%, Chi^2^ test* p* = 0.97) was found.

#### 3.3.3. Reduction of WC, HC, and WHR

**Figure 4:**
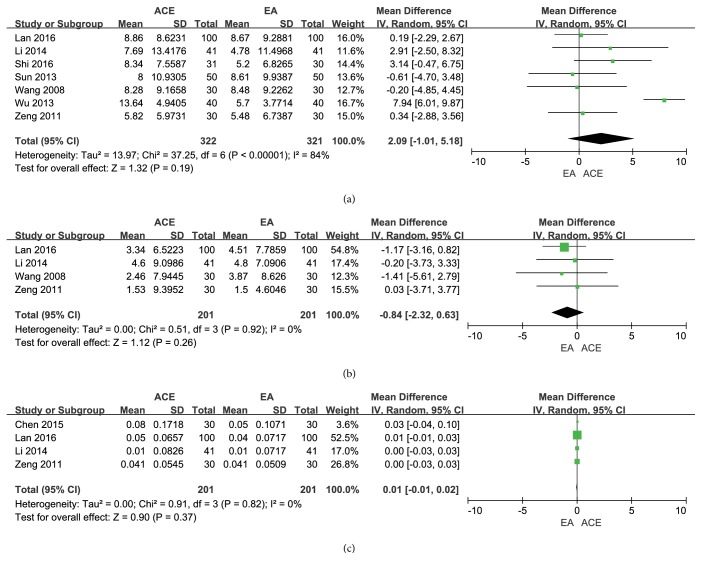


The combined reduction of WC from 7 trials [17, 23–26, 28, 29] had no significant difference between the two groups (MD = 2.09, 95%CI = -1.01~5.18,* p* = 0.19), and significant heterogeneity was found (*I*^2^ = 84%, Chi^2^ test* p* < 0.000001), as shown in Figure 4(a). Meanwhile, ACE was not superior to EA according to the pooled outcome of HC (MD = -0.84, 95%CI = -2.32~0.63,* p* = 0.26) of 4 trials [23, 24, 28, 29]; no significant heterogeneity (*I*^2^ = 0%, Chi^2^ test* p* = 0.92) was found in Figure 4(b). In addition, the pooled effect on WHR outcome in 4 records [22–24, 28] showed no significant difference in WHR decrease (MD = 0.01, 95%CI = -0.01~0.02,* p* = 0.37), and the heterogeneity is low (*I*^2^ = 0%, Chi^2^ test* p* = 0.82) (Figure 4(c)).

### 3.4. Comparison: ACE versus Sham

#### 3.4.1. BW and BMI

**Figure 5:**
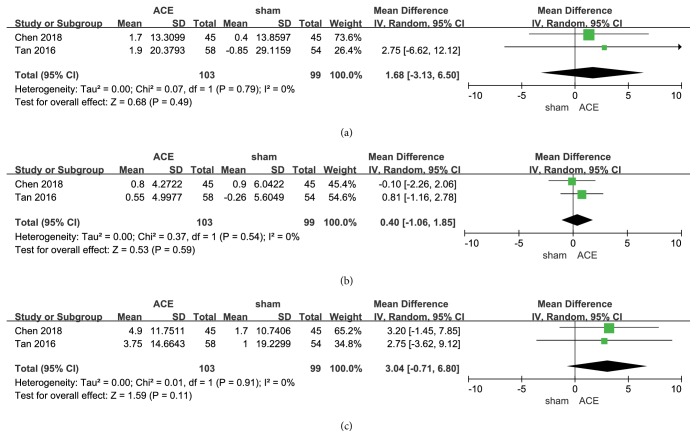


There are two trials [16, 21] reporting the reduction of BW, and no significant difference between the two interventions was found from the combined results indicated (MD = 1.68, 95%CI = -3.13~6.50,* p* = 0.49). It was considerably heterogeneous among the 2 studies (*I*^2^ = 0%, Chi^2^ test* p* = 0.79) and might result from the loss of embedding compared with ACE groups (Figure 5(a)).

The combined results of 2 studies [16, 21] showed that there was no significant difference about the reduction of BMI between the intervention of ACE and sham (MD = 0.40, 95%CI = -1.06~1.85,* p* = 0.59). No significant heterogeneity between the studies (*I*^2^ = 0%, Chi^2^ test* p* = 0.54) was found, as shown in Figure 5(b).

#### 3.4.2. Reduction of WC

There were 2 records [16, 21] reporting the reduction of WC in the comparison of ACE versus sham. There was no difference by their combination (MD = 3.04, 95%CI = -0.71~6.80,* p* = 0.11) and no heterogeneity between the results (*I*^2^ = 0%, Chi^2^ test* p* = 0.91) (Figure 5(c)).

### 3.5. Comparison: ACE Plus EA versus EA

#### 3.5.1. Frequency of Improvement

**Figure 6:**
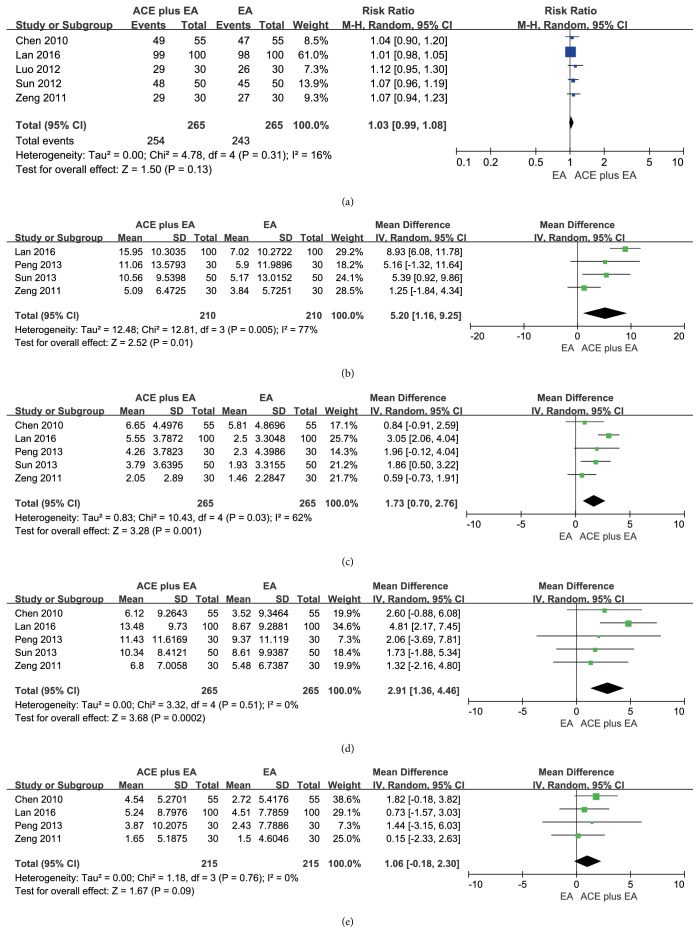


There was a significant difference in frequency of improvement according to the combined results of 5 studies [19, 20, 23, 27, 28] comparing the ACE plus EA with EA (RR = 1.03, 95% CI = 0.99~1.08,* p* = 0.13). There was no heterogeneity between the results (*I*^2^ =16%, Chi^2^ test* p* = 0.31) (Figure 6(a)).

#### 3.5.2. Reduction of BW and BMI

The combined results of 4 trials [18, 23, 25, 28] released the idea that ACE plus EA was better than EA alone statistically in the reduction of BW (MD = 5.20, 95%CI = 1.16~9.25,* p* = 0.01). Substantial heterogeneity between the results was found (*I*^2^ = 77%, Chi^2^ test* p* = 0.005) and may explain the difference of patients or acupoint prescription as shown in Figure 6(b).

Significant difference of BMI reduction (MD = 1.73, 95%CI = 0.70~2.76,* p* = 0.001) was tested by the pooled results of 5 studies [18, 20, 23, 25, 28]. There was obvious heterogeneity among the results (*I*^2^ = 62%, Chi^2^ test* p* = 0.03) (Figure 6(c)).

#### 3.5.3. Reduction of WC and HC

The studies [18, 20, 23, 25, 28] reported significant difference in WC loss between the two interventions (MD = 2.91, 95%CI = 1.36~4.46,* p* = 0.0002), and no heterogeneity was observed (*I*^2^ = 0%, Chi^2^ test* p* = 0.51) (Figure 6(d)). Four studies [18, 20, 23, 28] indicated that there was no difference in HC loss between the two interventions (MD = 1.06, 95%CI = -0.18~2.30,* p* = 0.0002), and heterogeneity was shown (*I*^2^ = 0%, Chi^2^ test* p* = 0.76), maybe caused by differences of patients or acupoint prescriptions (Figure 6(e)).

### 3.6. Adverse Events

There were two studies reporting adverse events in the comparison of ACE with EA. Wang [29] reported fainting during the treatment of ACE (n=1), as well as the treatment of EA (n=1). Shi [17] reported fainting (n=1), subcutaneous indurations (n=2), hematoma, and bruise (n=2) in ACE group. In addition, fainting, hematoma, and bruise were also observed in EA group with one case and three cases, respectively, along with sticking of the needle (n=1) during the treatment of EA.

### 3.7. Treatment Suggestion

**Figure 7:**
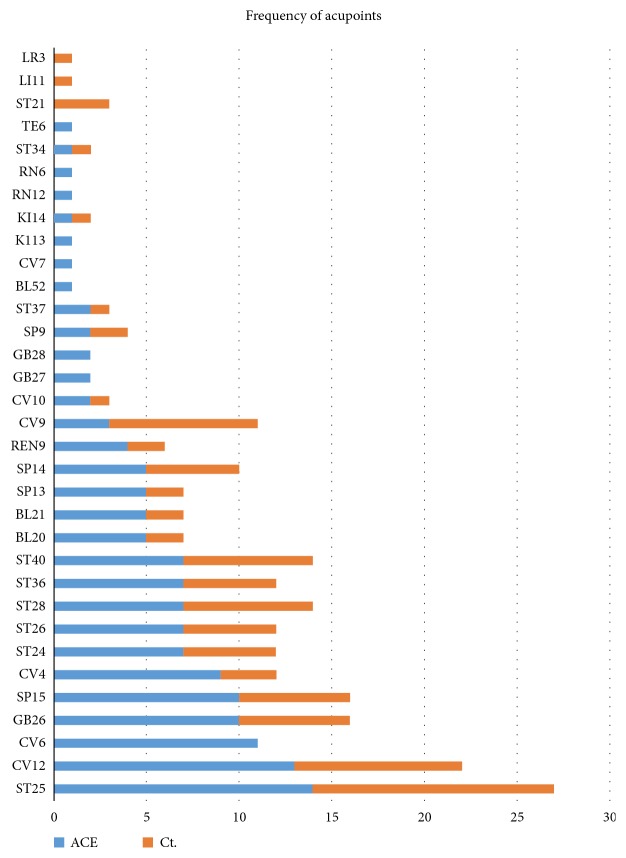


A total of 33 acupoints were extracted from the included RCTs, of which 30 acupoints were used in ACE group, and 24 were applied in control groups (EA, MA or sham). The frequency of usage was illustrated in Figure 7. Apparently, the acupoints of ST25, CV12, GB26, SP15, ST28, ST40 were the most used to lose weight both in ACE and control groups. In the ACE group, the frequency of treatment time ranged from 1 per 10 days to 2 per week, and most of the frequencies were 1 per week. Meanwhile, the total times of treatment varied from 4 weeks to 3 months, and 8 weeks was mostly used.

## 4. Discussions

Many studies have confirmed the negative impact of abdominal obesity on health, and it was reported to relate with cardiovascular disease [4], Alzheimer's disease, as well as other metabolic and vascular diseases [30]. ACE is efficient to control weight, but a systematic review of the efficiency of ACE in contrast with other types of acupuncture intervention for abdominal obesity appears to be lacking. In this review, a total of 15 RCTs including 1584 patients were selected and the efficiency of ACE was evaluated by comparing with MA, EA, and diet as the control.

Although the overall quality of included studies was poor owing to the poor methodological quality, small sample size and clinical heterogeneity, our meta-analysis had two key findings: (1) the pooled results suggested that ACE showed equal clinically effect comparing with MA or EA in losing weight and improving BMI for abdominal obesity. (2) A combination of ACE and EA (or MA) was more efficient than MA (or EA) alone for abdominal obesity.

Catgut embedding acupuncture is an updated and improved form of classic manual acupuncture with the advantages of lowering expense and time, as well as longer lasting stimulation without additional biological effect in comparison with manual acupuncture. In previous studies, RCTs manifested ACE was useful to reduce BW and improve the quality of life with less adverse effects [31–34], and some systematical reviews also reported that the effects of ACE on obesity were greater than or equal to other kinds of acupuncture. Specifically, a meta-analysis also suggested that ACE had better efficiency than MA or EA for simple obesity [35]. However, for abdominal obesity in this review, ACE showed no difference in losing weight in comparison with MA or EA, but the combination of ACE and EA (or MA) was superior to EA or MA alone which was consistent with other studies. It suggested that the efficiency of ACE might be type-specific for obesity in spite of being moderate overall effects. It may result from the response of different interventions to the various etiology of obesity.

Nowadays, many factors are suggested to be the etiology of obesity, such as neuromodulation, free radical, and genetics [36, 37]. In ancient TCM theory, it is believed that the dysfunction of spleen and stomach is the essential reason for obesity. The increasing intake of sweet and greasy foods and the function decline of spleen and stomach can lead to the accumulation of fat in the body. Obesity patients often suffer from the syndrome of* qi* deficiency and phlegm retardation [38, 39]. It was proved that the blood pressure, blood sugar, cholesterol, and triglyceride of abdominal obesity patients were higher than those of the non-abdominal obesity patients, whereas the HDL cholesterol level was significantly lower than that of the control group [40]. Abdominal obesity is closely related to metabolic abnormalities and the control of abdominal obesity contributes to the early prevention of metabolic syndrome and cardiovascular diseases.

There were several limitations in this review: (1) It is hard to evaluate the safety of ACE because of the lack of adverse effect data in primary studies. (2) Owing to the relatively short duration of ACE sessions and follow-up duration of included studies, there are unaddressed clinical concerns with respect to the long-term effects of ACE on weight control. (3) The quality of many analyzed RCTs was unsatisfactory with unclaimed details of randomization, blinding methods and so on. Therefore, further clinical trials with rigorous design and longer follow-up appear warranted.

## 5. Conclusions

Our review found the evidence that the effects of abdominal obesity treated by ACE were superior or equal to other interventions (MA, EA, and diet) based on the assessment of the pooled outcomes (frequency of improvement, loss of BW, BMI, WC, and HC), whereas the combination of ACE and EA or MA is more effective than EA or MA alone. Further studies with rigorous design are required to overcome the limitations of small sample size and short-term effect and evaluate the effect of ACE for treating abdominal obesity.

## Figures and Tables

**Table 1:** Characteristics of RCTs of ACE for abdominal obesity.

First Author, year	No. in ACE/Ct.	Intervention					Outcome (ACE versus Ct.)		Side effects	
		Acupoints of ACE	Type of Ct.	Frequency of ACE	Frequency of Ct.	Acupoints of Ct.	With benefit	No effect	ACE	EA
Tan et al, 2016	58/54	CV12, ST25, SP15, SP9, GB26, ST40	sham	1 per wk (4 wks)	1 per wk (4 wks)	CV12, ST25, SP15, SP9, GB26, ST40	BW, BMI, WC, Thickness of subcutaneous fat in the renal capsule, left and right parumbilical region	Subumbilical epithelial fat thickness, pre-hepatic fat thickness	/	/
Lan et al, 2016	100/100	CV12, CV6, ST24, ST26, SP13, SP15, BL20, BL21, REN9, CV4, ST25, ST28, SP14, GB26, ST36, ST40	EA	1 per wk (12 wks)	3 per wk (12 wks)	ST24, ST26, ST25, SP14	/	BW, WC, HC, BMI, WHR	/	/
Chen et al, 2015	30/30	GB26, ST25, ST28, CV4, GB27, GB28, K113, BL52	EA	1 per wk (8 wks)	1 per 2 days (8 wks)	CV12, GB26, ST36, ST25, ST40, ST28	WHR, WHtR, BMI, body fat ratio	/	/	/
Deng et al, 2014	30/30	CV12, ST25, CV7, TE6, CV4, ST36	Diet	1 per wk (3 mths)	1 per 3 days (3 mths)	/	BW, WC	/	/	/
Li et al, 2014	41/41	GB26, ST25, SP15, RN12, RN6	EA	1 per wk (8 wks)	1 per 2 days (8 wks)	GB26, SP15, ST25, CV12, CV6, ST21, ST28, ST36, ST40	/	BW, WC, HC, BMI, WHtR, body fat ratio	/	/
Sun et al, 2013	50/50	CV12, CV6, ST24, ST26, SP13, SP15, BL20, BL21, REN9, CV4, ST25, ST28, SP14, GB26, ST36, ST40	EA	1 per wk (8 wks)	3 per wk (8 wks)	CV12, CV6, ST24, ST26, SP13, SP15, BL20, BL21, REN9, CV4, ST25, ST28, SP14, GB26, ST36, ST40	ISI, IRI, HDL	BW, WC, BMI, FPG, FINS, CH, TG, LDL	/	/
Wu et al, 2013	40/40	CV12, CV10, CV6, CV4, ST34, ST25	EA	1 per 10 days (100 days)	5 per wk	CV12, CV10, CV6, CV4, ST34, ST25	BW, WC	/	/	/
Sun et al, 2012	50/50	CV12, CV6, ST24, ST26, SP13, SP15, BL20, BL21, REN9, CV4, ST25, ST28, SP14, GB26, ST36, ST40	EA	1 per wk (8 wks)	3 per wk (8 wks)	ST24, ST26, ST25, SP14	BW, WC	/	/	
Zeng et al, 2011	30/30	CV12, CV6 ST24, ST26, SP13, SP15 BL20, BL21, REN9, CV4, ST25, ST28, SP14, GB26, ST36, ST40, CV12, CV6, ST24, ST26, SP15	EA	1 per wk (12 wks)	3 per wk (12 wks)	CV12, CV6, ST24, ST26, SP13, SP15, BL20, BL21, REN9, CV4 ST25, ST28, SP14, GB26, ST36, ST40	BMI	BW, WC, HC, WHR	/	/
Wang et al, 2008	30/30	CV12, ST25, CV6, ST37	EA	1 per 10 days (3 mths)	1 per 2 days (3 mths)	ST21, ST25, SP15, ST28	/	BW, WC, HC, chest circumference, Upper arm circumference, thigh circumference, calf circumference, BMI	fainting	fainting
Chen et al, 2018	40/40	CV6, CV9, ST28, KI14, ST36	Sham	2 per wk (6 wks)	2 per wk (6 wks)	CV6, CV9, ST28, KI14, ST36	BW, WC, HC, BMI, Diastolic blood pressure	Systolic blood pressure, Heart rate,	/	/
Luo et al, 2012	30/30	CV12, ST25, SP15, CV6, ST37	EA	1 per 10 days (3 mths)	1 per 2 days (3 mths)	CV12, ST21, ST25, SP15, ST28, CV6, ST37	/	/		
Peng et al, 2013	30/30	CV12, CV6, ST24, ST26, SP13, SP15, BL20, BL21, SP9, CV9, CV4, ST25, ST28, SP14, GB26, ST36, ST40	EA	1 per wk (8 wks)	3 per wk (8 wks)	ST24, ST26, ST25, SP14	/	/	/	/
Chen et al, 2010	55/55	GB26, CV12, ST25, CV9, ST40	EA	1 per wk (8 wks)	5 per wk (8 wks)	GB26, CV12, ST25, CV9, ST40	/	/	/	/
Shi et al, 2016	31/30	CV12, CV10, CV6, CV4, ST24, ST26, SP15, GB26, ST25, GB27, GB28	EA	1 per wk (8 wks)	3 per wk (8 wks)	LI11, ST25, CV12, SP9, ST40, LR3	/	/	subcutaneous indurations, hematoma, bruise, fainting	sticking of needle, hematoma, bruise, fainting

## References

[1] Cawley J., Meyerhoefer C. (2012). The medical care costs of obesity: an instrumental variables approach. *Journal of Health Economics*.

[2] Park B.-Y., Lee M. J., Kim M., Kim S.-H., Park H. (2018). Structural and functional brain connectivity changes between people with abdominal and non-abdominal obesity and their association with behaviors of eating disorders. *Frontiers in Neuroscience*.

[3] Expert Panel on Detection Evaluation and Treatment of High Blood Cholesterol in Adults (2001). Executive summary of the third report of the National Cholesterol Education Program (NCEP) expert panel on detection, evaluation, and treatment of high blood cholesterol in adults (adult treatment panel III). *Journal of the American Medical Association*.

[4] Yusuf S., Hawken S., Ounpuu S. (2004). Effect of potentially modifiable risk factors associated with myocardial infarction in 52 countries (the INTERHEART study): Case-control study. *The Lancet*.

[5] Ritchie S. A., Connell J. M. C. (2007). The link between abdominal obesity, metabolic syndrome and cardiovascular disease. *Nutrition, Metabolism & Cardiovascular Diseases*.

[6] Després J.-P., Lemieux I. (2006). Abdominal obesity and metabolic syndrome. *Nature*.

[7] Salehinia F., Abdi H., Hadaegh F. (2018). Abdominal obesity phenotypes and incident diabetes over 12 years of follow-up: The Tehran Lipid and glucose study. *Diabetes Research and Clinical Practice*.

[8] Fang S., Wang M., Zheng Y., Zhou S., Ji G. (2017). Acupuncture and lifestyle modification treatment for obesity: a meta-analysis. *American Journal of Chinese Medicine*.

[9] Du H.-Y., Zhang Y.-B. (2013). The effect and molecular mechanism of acupuncture and moxibustion in treating obesity. *Shanghai Journal of Acupuncture and Moxibustion*.

[10] Sui Y., Zhao H. L., Wong V. C. W. (2012). A systematic review on use of chinese medicine and acupuncture for treatment of obesity. *Obesity Reviews*.

[11] Gong M., Wang X., Mao Z., Shao Q., Xiang X., Xu B. (2012). Effect of electroacupuncture on leptin resistance in rats with diet-induced obesity. *American Journal of Chinese Medicine*.

[12] Gong M., Cao C., Chen F. (2016). Electroacupuncture attenuates hepatic lipid accumulation via AMP-activated protein kinase (AMPK) activation in obese rats. *Acupuncture in Medicine*.

[13] Deng L., Lun Z., Ma X., Zhou J. (2014). Clinical observation on regulating the three energizer by acupoint catgut embedding combined with abdominal acupuncture in treating abdominal obesity: a randomized controlled trial. *World Journal of Acupuncture - Moxibustion*.

[14] Guo T., Ren Y., Kou J. (2015). Acupoint catgut embedding for obesity: systematic review and meta-analysis. *Evidence-Based Complementary and Alternative Medicine*.

[15] Higgins J.-P.-T., Green S. (2011). *Cochrane Handbook for Systematic Reviews of Interventions Version 5.1.0*.

[16] Tan W.-L. (2016). Clinical study on abdominal type of simple onesity treated with acupoint catgut embedding threapy. *Beijing Journal of Traditional Chinese Medicine*.

[17] Shi Y.-Y. (2016). *Clinical Efficacy of Abdominal Acupoint Catgut Embedding Treatment of Abdominal Obesity*.

[18] Peng Y.-F. (2013). *Clinical Study of Electroacupuncture and Catgut Embedding on Spleen Deficiency and Dampness Stagnation Abdominal Obesity in the Quality of Life*.

[19] Luo Y. (2012). Efficacy of acupuncture combined with acupoint catgut embedding in the treatment of metabolic syndrome. *Chinese Journal of Gerontology*.

[20] Chen X.-X. (2010). *Clinical Effect Observation of Treating Gentral Obesity Wth Electric Neddle Acpuncture plus Catgut Embedding*.

[21] Chen I., Yeh Y., Hsu C. (2018). Therapeutic effect of acupoint catgut embedding in abdominally obese women: a randomized, double-blind, placebo-controlled study. *Journal of Women's Health*.

[22] Chen Z.-J. (2015). Clinical effect observation of treating abdominal obesity with vein acupoint catgut embedding. *Chinese Manipulation & Rehabilitation Medicine*.

[23] Lan D.-C. (2016). Randomized controlled trial of electro-acupuncture combined with acupoint thread-embedding for treatment of abdominal obesity. *Journal of Guangzhou University Traditional Chinese Medicine*.

[24] Li Y.-Y. (2014). Therapeutic observation of acupoint thread-embedding for abdominal obesity. *Shanghai Journal of Acupuncture and Moxibustion*.

[25] Sun J. (2013). Effect of electroacupuncture plus acupoint thread-embedding on glucose and lipid metabolism in abdominal obesity patients. *Shanghai Journal of Acupuncture and Moxibustion*.

[26] Wu Y.-Y., Liang Q.-X., Liang W.-Q. (2013). Clinical effect observation of acupoint catgut embedding in treatment of abdominal obesity. *Medical Infornation*.

[27] Sun J. (2012). Clinical study on improving the quality of life of patients with abdominal obesity by electroacupuncture combined with acupoint catgut embedding. *Journal of New Chinese Medicine*.

[28] Zeng Z.-P. (2011). *Acupuncture, Catgut Embedding Therapy Clinical Study of Simple Central Obesity*.

[29] Wang Q. (2008). *Clinical Analysis on Acupoint Catgut-Embedding Therapy for Abdominal Obesity*.

[30] Razay G., Vreugdenhil A., Wilcock G. (2006). Obesity, abdominal obesity and alzheimer disease. *Dementia and Geriatric Cognitive Disorders*.

[31] Xu Z., Li R., Zhu C., Li M. (2013). Effect of acupuncture treatment for weight loss on gut flora in patients with simple obesity. *Acupuncture in Medicine*.

[32] Rerksuppaphol L. (2012). Efficacy of auricular acupressure combined with transcutaneous electrical acupoint stimulation for weight reduction in obese women. *Journal of the Medical Association of Thailand*.

[33] Gucel F., Bahar B., Demirtas C., Mit S., Çevik C. (2012). Influence of acupuncture on leptin, ghrelin, insulin and cholecystokinin in obese women: a randomised, sham-controlled preliminary trial. *Acupuncture in Medicine*.

[34] Hsu C.-H., Hwang K.-C., Chao C.-L., Lin J.-G., Kao S.-T., Chou P. (2005). Effects of electroacupuncture in reducing weight and waist circumference in obese women: a randomized crossover trial. *International Journal of Obesity*.

[35] Liao J.-Q., Song X., Chen Y., Liang L.-C., Wang S.-X. (2014). Clinical randomized controlled trials of acupoint catgut-embedding for simple obesity: a meta-analysis. *Chinese acupuncture & moxibustion*.

[36] Yamamoto J., Imai J., Izumi T. (2017). Neuronal signals regulate obesity induced ${\hat{I}}^{2}$-cell proliferation by FoxM1 dependent mechanism. *Nature Communication*.

[37] Bouter K. E., van Raalte D. H., Groen A. K., Nieuwdorp M. (2017). Role of the gut microbiome in the pathogenesis of obesity and obesity-related metabolic dysfunction. *Gastroenterology*.

[38] Quan X. L., Duan J. (2010). Viewpoint of obesity. *Journal of TongJi University (Medical Science)*.

[39] Gong H.-Y., Zhang H.-M., Wang R.-L. (2004). Cognition of obesity in ancient doctors. *Beijing Journal of Traditional Chinese Medicine*.

[40] Sun Y. J. (2009). The relationship between central obesity and metabolic abnormality. *Mod Journal of Integrated Traditional Chinese West Medicine*.

